# Structural insight into the catalytic mechanism of the bifunctional enzyme l-fucokinase/GDP-fucose pyrophosphorylase

**DOI:** 10.1016/j.jbc.2025.108344

**Published:** 2025-02-22

**Authors:** Sheng-Wei Lin, Tzu-Ping Ko, Hung-Yu Chiang, Cheng-Guo Wu, Min-Feng Hsu, Andrew H.-J Wang, Chun-Hung Lin

**Affiliations:** 1 Institute of Biological Chemistry, Academia Sinica, Taipei, Taiwan; 2 Institute of Biochemical Sciences, National Taiwan University, Taipei, Taiwan; 3 PhD. Program for Translational Medicine, College of Medical Science and Technology, Taipei Medical University, Taipei, Taiwan; 4 Department of Chemistry, National Taiwan University, Taipei, Taiwan

**Keywords:** *Bacteroides fragilis*, bifunctional, carbohydrate biosynthesis, enzyme mechanism, GDP-fucose pyrophosphorylase, glycobiology, l-fucokinase, protein structure, X-ray crystallography

## Abstract

The bifunctional l-fucokinase/GDP-**β**-l-fucose pyrophosphorylase (FKP) from *Bacteroides fragilis* catalyzes the conversion from l-fucose to GDP-**β**-l-fucose. The reaction product, representing the activated form of l-fucose, is used by all l-fucosyltransferases to incorporate l-fucose. Herein, we report the first X-ray crystal structures of FKP in complex with substrate–product, leading to the dissection of both activity domains and corresponding catalytic mechanisms. The full-length FKP (FKP-FL, 949 amino acids) exists as a tetramer in solution, but the individually prepared N-terminal domain (FKP-NTD corresponding to the sequence 1–496, also containing a SUMO tag) and C-terminal domain (FKP-CTD, the sequence 519–949) form a monomer and a dimer, respectively. FKP-NTD has a single **α/β** domain and a **β-**helix-containing domain, whereas FKP-CTD folds into two **α/β** domains and the linker comprises three **α**-helices. The **β**-l-fucose-1-phosphate (fucose-1-P) and GTP bound separately to the active sites of fucokinase (located at FKP-CTD) and pyrophosphorylase (FKP-NTD), and a third nucleotide-binding site is adjacent to the **β**-helix (also in FKP-NTD). Furthermore, Asp762 was proposed to serve as the general base in the reaction of fucokinase, to deprotonate the C1-OH of fucose in the nucleophilic attack to **γ**-phosphate of ATP, resulting in the formation of fucose-1-P. At the same time, Arg592 and magnesium ion stabilize the developing negative charge in the leaving group (ADP). Subsequently, in the pyrophosphorylase-catalyzed reaction, the Lys187 side chain facilitates the nucleophilic attack of fucose-1-P toward GTP, leading to the formation of GDP-fucose.

l-Fucose is an important monosaccharide decorating the nonreducing end of oligosaccharides and glycan chains of glycoproteins and glycolipids (1). Fucosylation, the incorporation of l-fucose, is involved in various biological processes. For instance, Lewis antigens (*e.g.*, sialyl Lewis x and sialyl Lewis a) represent an important class of fucosylated glycans existing on the surface of mammalian cells (2, 3). They are known to serve as the ligands of selectins in inflammatory response (4, 5), cell–cell adhesion (6), and tumorigenesis (7, 8). A number of reports indicated that fucosylation is critical to the processes of fertilization (9), development (10, 11), and apoptosis (12, 13). Fucosylated glycans have been recognized as an invading strategy for pathogenic bacteria to either avoid the host immune surveillance by mimicking host polysaccharides or mediate the host–microbe adhesion (14, 15, 16). L-Fucosyltransferases catalyze the incorporation of l-fucose into the glycan chains from the donor substrate: GDP-β-l-fucose (GDP-fucose), the activated form of l-fucose (14). There are two pathways to synthesize GDP-fucose *in vivo*, including the *de novo* and salvage pathways (17). The *de novo* pathway employs two enzymes for converting GDP-α-d-mannose to GDP-fucose: GDP-α-d-Man-4,6-dehydratase and GDP-4-keto-6-deoxy-d-Man-3,5-epimerase-4-reductase (18, 19). The salvage pathway also utilizes two enzymes. l-fucokinase (Enzyme Commission number: 2.7.1.52) catalyzes the phosphorylation of l-fucose with ATP, forming β-l-fucose-1-phosphate (fucose-1-P). GDP-fucose pyrophosphorylase (Enzyme Commission number: 2.7.7.30) is responsible for the further condensation of fucose-1-P with GTP to produce GDP-fucose (20, 21). The bifunctional enzyme, l-fucokinase/GDP-fucose pyrophosphorylase, was isolated from *Bacteroides fragilis* 9343 (Fig. 1). FKP (l-fucokinase/GDP-β-l-fucose pyrophosphorylase) was previously reported to participate in the biosynthesis of bacterial capsular polysaccharides, thus offering *B. fragilis* a survival advantage in competitive host intestinal ecosystem (22).

**Figure 1:**
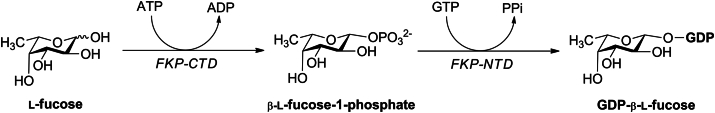
**The bifunctional protein FKP from *Bacteroides fragilis* 9343 is responsible for the biosynthesis of GDP-fucose.** The C-terminal domain (FKP-CTD) catalyzes the formation of fucose-1-P from l-fucose and ATP, whereas the N-terminal domain (FKP-NTD) catalyzes the condensation of fucose-1-P with GTP to produce GDP-fucose. CTD, C-terminal domain; NTD, N-terminal domain.

Furthermore, enzymatic fucosylation is frequently utilized in enzymatic synthesis of carbohydrates, with GDP-fucose serving as the universal donor substrate for l-fucosyltransferases (23, 24, 25). One major hurdle, nevertheless, is the high cost and instability of GDP-fucose (26). Applying *B. fragilis* FKP for the GDP-fucose synthesis has been shown a practical and efficient way for enzymatic fucosylation. In addition, 5-azide- or 5-alkyne-substituted l-fucose has been frequently employed for specific labeling of fucosylated glycans (together with the use of click-chemistry for fluorescent labeling), taking advantage of the *in vivo* salvage pathway (16, 27). This FKP is known to be flexible toward the C-5 position of l-fucose (26), but the molecular basis of such relaxed specificity has not been shown. Several structural determination studies were reported to provide substantial information (28, 29, 30). Especially, the cryo-EM work by Liu *et al.* (30) elucidated the quaternary structure and identified several residues critical to the enzyme activity. Further understanding is, however, limited by the lack of high-resolution structures of FKP in complex with substrate or product. Herein, we present for the first time X-ray crystallographic FKP complex structures with in-depth biophysical and enzymatic characterizations. The results clearly demonstrate the catalytic mechanisms of FKP and provide a basis for the design of GDP-fucose derivatives.

## Results and discussion

### The main regions and oligomeric status of FKP

There are 949 amino acids in *Bacteroides fragils* FKP. The analysis of sequence alignment indicates that the N terminus (amino acids 1–430) shares 23% identity with the human GDP-fucose pyrophosphorylase, whereas the C terminus (amino acids 584–949) shares 33% identity with the human l-fucokinase. Connecting the two domains was predicted a linker of 153-amino acid residues (corresponding to the sequence 431–583), but the function is unknown (31). Since the three-dimensional structure of FKP is not yet available at the time the experiments were initiated, systemic domain-based truncations with different lengths were performed to define the main regions of N temini, C termini, and the linker. The chimeric DNA inserts were sequenced to assure no mutation; all the clones were expressed under the same condition. After cell disruption and centrifugation, the initial protein expression screening was analyzed by SDS-PAGE and Western blotting analysis (data not shown). The schematic diagram depicted the protein solubility of full-length (FL) and different truncated FKPs (Fig. S1). Deletions of N-terminal 42 and 99 amino acids resulted in formation of insoluble proteins, suggesting that this region is critical to the protein structure. The result showed that N-terminal activity domain needs a minimum number of 496 amino acid residues but not exceeds 597 (Fig. S1). The optimal lengths of C-terminal main region are from amino acid 518 or 548 to 949. The region of connecting these two domains also was studied from different deletions of 49 to 155 residues, but none of these truncated proteins were soluble. It is shown that the linker region is also essential for FKP because it not only serves as a linker but also plays a critical role in stabilizing the protein structure or interacting with each of the two domains. The FL (amino acid sequence 1–949), N-terminal domain (NTD;1–496), and C-terminal domain (CTD; 518–949) were therefore individually prepared and designated as FKP-FL, FKP-NTD, and FKP-CTD, respectively.

**Figure 2:**
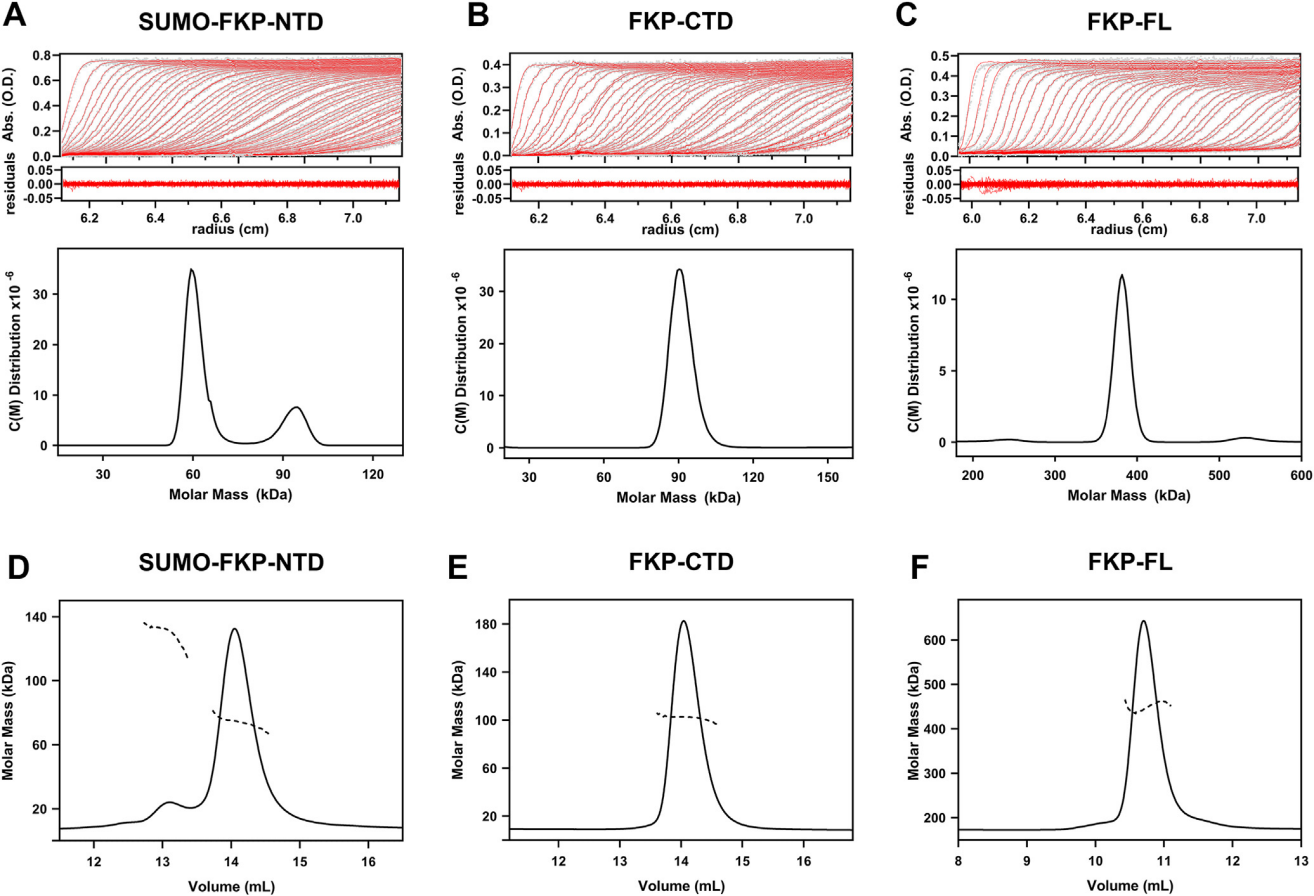
**Molecular weights of FKPs determined by SV-AUC and SEC–MALS analysis.** The SV–AUC datasets are shown for SUMO-FKP-NTD (*A*), FKP-CTD (*B*), and FKP-FL (*C*). The UV absorbance data with residuals were fitted with continuous c(M) distribution using the Sedfit program. SEC–MALS chromatograms (*D*–*F*) correspond to SUMO-FKP-NTD, FKP-CTD, and FKP-FL, respectively, with light-scattering signals plotted and fitted to exhibit the calculated molar masses (*dotted lines*). AUC, analytical ultracentrifugation; CTD, C-terminal domain; FL, full length; MALS, multiangle light scattering; NTD, N-terminal domain; SEC, size-exclusion chromatography; SV, sedimentation velocity.

The oligomeric status of purified FKPs was examined by gel-filtration chromatography, size-exclusion chromatography–multiangle light scattering (SEC–MALS), and analytical ultracentrifugation (AUC) analysis. Both gel-filtration chromatography and sedimentation velocity (SV) experiments of AUC showed that both FKP-FL and FKP-CTD exist as a single species in solution, whereas FKP-NTD appeared as multipatterned oligomers with the molecular weight (MW) ranging from 50 to 500 kDa even though the protein was freshly prepared before the analysis (data not shown). Several conditions of expression and purification were thus examined to solve the problem. Finally, FKP-NTD fused with a SUMO tag protein displayed greatly improved homogeneity despite a small fraction of the protein mixture still forming a larger MW (Fig. 2*A*). According to the SV–AUC analysis, SUMO-FKP-NTD, FKP-CTD, and FKP-FL were estimated to contain the MWs of 62, 95, and 390 kDa (Fig. 2, *A*–*C*). Likewise, the SEC–MALS analysis of SUMO-FKP-NTD, FKP-CTD, and FKP-FL indicated the MWs of 74, 100, and 420 kDa, respectively (Fig. 2, *D*–*F*). The protein sequences of SUMO-FKP-NTD, FKP-CTD, and FKP-FL were calculated to have the MWs of 69, 49, and 108 kDa, respectively. Therefore, SUMO-FKP-NTD is primarily a monomeric protein. FKP-CTD forms a dimer, whereas FKP-FL exists as a tetramer in solution.

### Enzymatic properties of FKP

The initial activity screening was performed by TLC analysis to check if these chimeric domains are active or functional. The results indicated that FKP-NTD only has GDP-fucose pyrophosphorylase activity to catalyze the reaction of fucose-1-P with GTP, leading to the formation of GDP-fucose. FKP-CTD contains l-fucokinase activity to catalyze the phosphorylation of l-fucose with ATP, resulting in the production of fucose-1-P. FKP-FL was found to have both enzyme activities to generate GDP-fucose (data not shown). Taken together, FKP has two distinct activities; GDP-fucose pyrophosphorylase and l-fucokinase activity are separated into two different activity domains and located at N termini and C termini, respectively.

**Figure 3:**
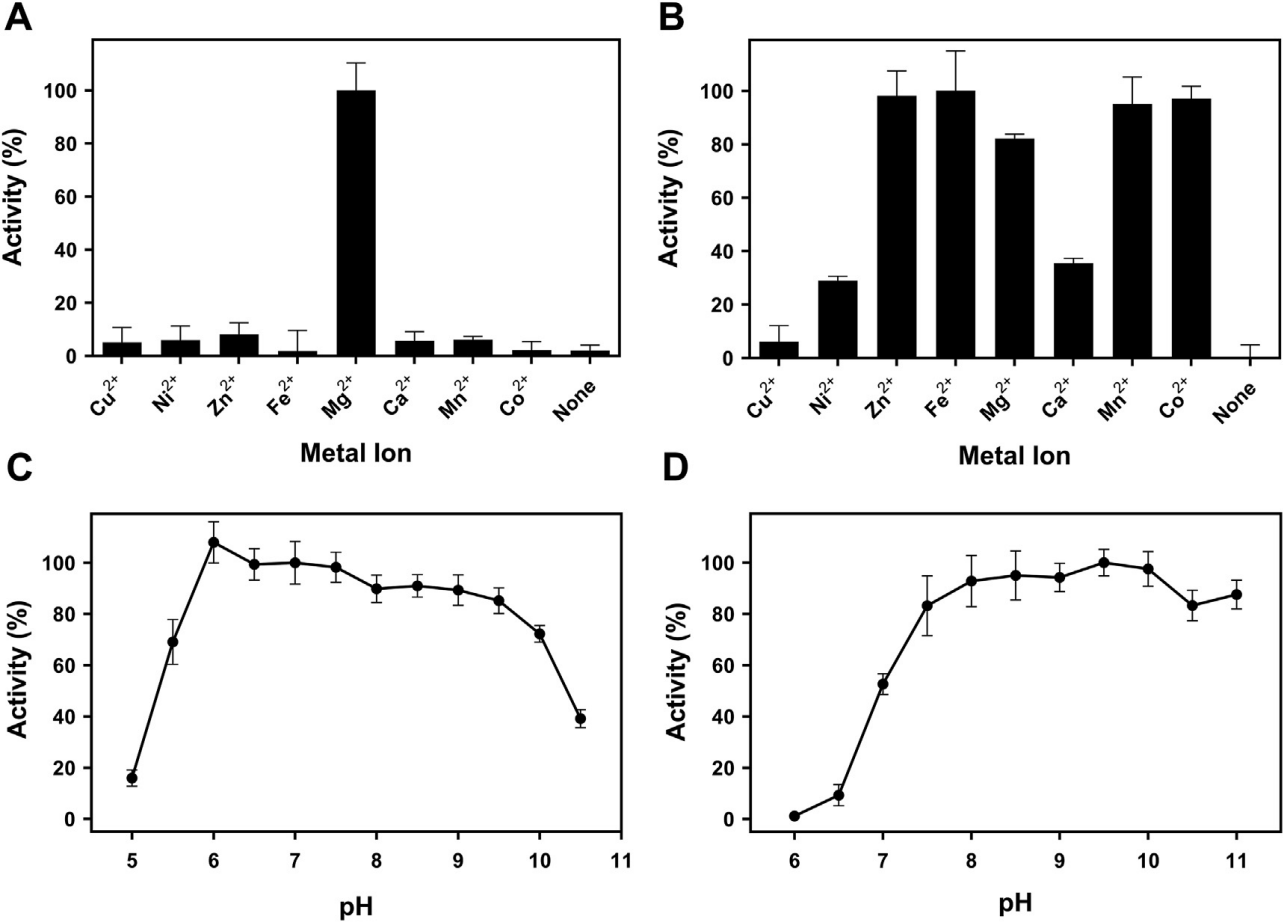
**Effects of metal ion and pH on the activities of GDP-fucose pyrophosphorylase and fucokinase.** FKP-FL (final concentration: 2 mM) was used for divalent metal ion screening of GDP-fucose pyrophosphorylase (*A*) and fucokinase (*B*). The optimal pH profiles of GDP-fucose pyrophosphorylase (*C*) and fucokinase (*D*) were obtained using 50 mM buffer of different pH values. Data are averages of triplicate assays. FL, full length.

We in addition surveyed the metal ion dependence and pH profile of both enzyme activities (Fig. 3). GDP-fucose pyrophosphorylase activity of FKP absolutely requires magnesium ion, and the resulting activity is much higher than those using other divalent metal ions. l-Fucokinase activity of FKP shows a broader selection for divalent metal ions, such as Co^2+^, Fe^2+^, Mg^2+^, Mn^2+^, and Zn^2+^. GDP-fucose pyrophosphorylase showed a typical pH profile from 6 to 9.5, but l-fucokinase appeared to prefer alkaline pH with a range between pH 7.5 and 11. Therefore, pH 7.5 was set for the activity assay of FKP throughout further studies.

### Substrate specificity of FKP

To investigate whether FKP accepts other monosaccharides for the kinase reaction in addition to l-fucose, several monosaccharides were examined in the presence of ATP. Among them, l-fucose, 5-azido-l-fucose, 5-alkynyl-l-fucose, d-arabinose, and l-galactose were found acceptable and phosphorylated to produce sugar-1-phosphates. Apparently, the enzyme is restricted to the stereochemical configurations at C2–C5 positions of monosaccharides but is flexible to C5-substituent of l-fucose. Compared with the nature substrate l-fucose (*K*_*m*_ = 34 μM), d-arabinose (2450 μM) and l-galactose (3345 μM) displayed reduced affinity about 70- and 100-fold, respectively. Moreover, the resulting sugar-1-phosphates were also acceptable for GDP-fucose pyrophosphorylase to produce GDP-fucose and analogs. d-Mannose-1-phosphate and d-glucose-1-phosphate (both commercially available) were also tested for the enzyme activity, but none of them were acceptable. GDP-l-fucose, GDP-d-arabinose, GDP-l-galactose, GDP-azido-l-fucose, and GDP-alkynyl-l-fucose were synthesized directly from the corresponding monosaccharides by FKP (see Fig. S2 for substrate screening and product characterization). These GDP-fucose derivatives presumably serve as powerful tools for dissecting the roles and functions of fucosylated glycans.

Furthermore, the kinetic parameters on fucokinase of FKP-CTD and FKP-FL were examined by measuring the activities with varying concentrations of l-fucose and ATP. As shown in Table 1, there was no apparent difference in *k*_cat_ values for fucokinase in FKP-CTD and FKP-FL. In comparison with FKP-FL, FKP-CTD increased 10-fold in *K*_*m*_ for l-fucose and twofold for ATP, resulting in 3∼15-fold decrease in the catalytic efficiency (*k*_cat_/*K*_*m*_). Interestingly, the activity *versus* ATP concentration plot showed a sigmoidal curve and clearly exhibited positively cooperative behavior on fucokinase for ATP, as indicated by Hill coefficients (Table 1). Meanwhile, the kinetic parameters of GDP-fucose pyrophosphorylase were also determined. FKP-FL exhibited higher affinities for both substrates in comparison with SUMO-FKP-NTD. SUMO-FKP-NTD showed significant decrease in the turnover number. Therefore, the FL protein offers much higher catalytic efficiency than the truncated domains.

**Table 1:** Analysis of kinetic parameters of FKP, domain fragments, and mutants

	Fucose-1-P			GTP		
FKPs	*K*_*m*_ (mM)	*k*_cat_ (s^−1^)	*k*_cat_/*K*_*m*_	*K*_*m*_ (mM)	*k*_cat_ (s^−1^)	*k*_cat_/*K*_*m*_
FKP-FL	0.040 ± 0.004	5.6 ± 0.3	140	0.008 ± 0.001	5.1 ± 0.2	638
SUMO-FKP-NTD	0.080 ± 0.008	0.42 ± 0.05	5.3	0.19 ± 0.03	0.33 ± 0.04	1.7
FKP-FL-D31A	0.081 ± 0.009	0.022 ± 0.008	0.27	0.047 ± 0.008	0.028 ± 0.004	0.60
FKP-FL-H73A	0.050 ± 0.007	0.32 ± 0.03	6.4	0.037 ± 0.008	0.41 ± 0.07	11.1
FKP-FL-R80A	0.20 ± 0.019	0.012 ± 0.003	0.062	0.051 ± 0.009	0.020 ± 0.008	0.39
FKP-FL-D136A	ND^a^			ND		
FKP-FL-H168A	0.72 ± 0.13	0.027 ± 0.006	0.038	0.040 ± 0.010	0.057 ± 0.01	1.43
FKP-FL-K187A	1.2 ± 0.2	0.15 ± 0.02	0.13	0.062 ± 0.008	0.17 ± 0.01	2.74
FKP-FL-H272A	0.53 ± 0.082	0.034 ± 0.05	0.064	0.030 ± 0.005	0.053 ± 0.002	1.77


	Fucose			ATP			
FKPs	*K*_*m*_ (mM)	*k*_cat_ (s^−1^)	*k*_cat_/*K*_*m*_	*K*_*m*_ (mM)	*k*_cat_ (s^−1^)	*k*_cat_/*K*_*m*_	h^b^
FKP-FL	0.034 ± 0.003	4.6 ± 0.2	135	1.8 ± 0.1	3.8 ± 0.19	2.1	1.4
FKP-CTD	0.33 ± 0.02	2.8 ± 0.2	8.6	4.4 ± 0.5	2.6 ± 0.23	0.6	2.0
FKP-FL-W599A	9.2 ± 0.81	0.30 ± 0.03	0.03	0.92 ± 0.2	0.28 ± 0.05	0.3	1.5
FKP-FL-D601A	8.2 ± 1.1	0.36 ± 0.03	0.04	3.4 ± 0.5	0.26 ± 0.04	0.08	1.2
FKP-FL-E751A	ND			ND			
FKP-FL-D762A	ND			ND			

The data of *K*_*m*_ and *k*_cat_ represent three independent experiments (mean ± SEM).

a ND, not detected (because of no or very low activity).

b h, Hill coefficient (analyzed by GraphPad Prism software).

### Crystal structures of FKP

**Figure 4:**
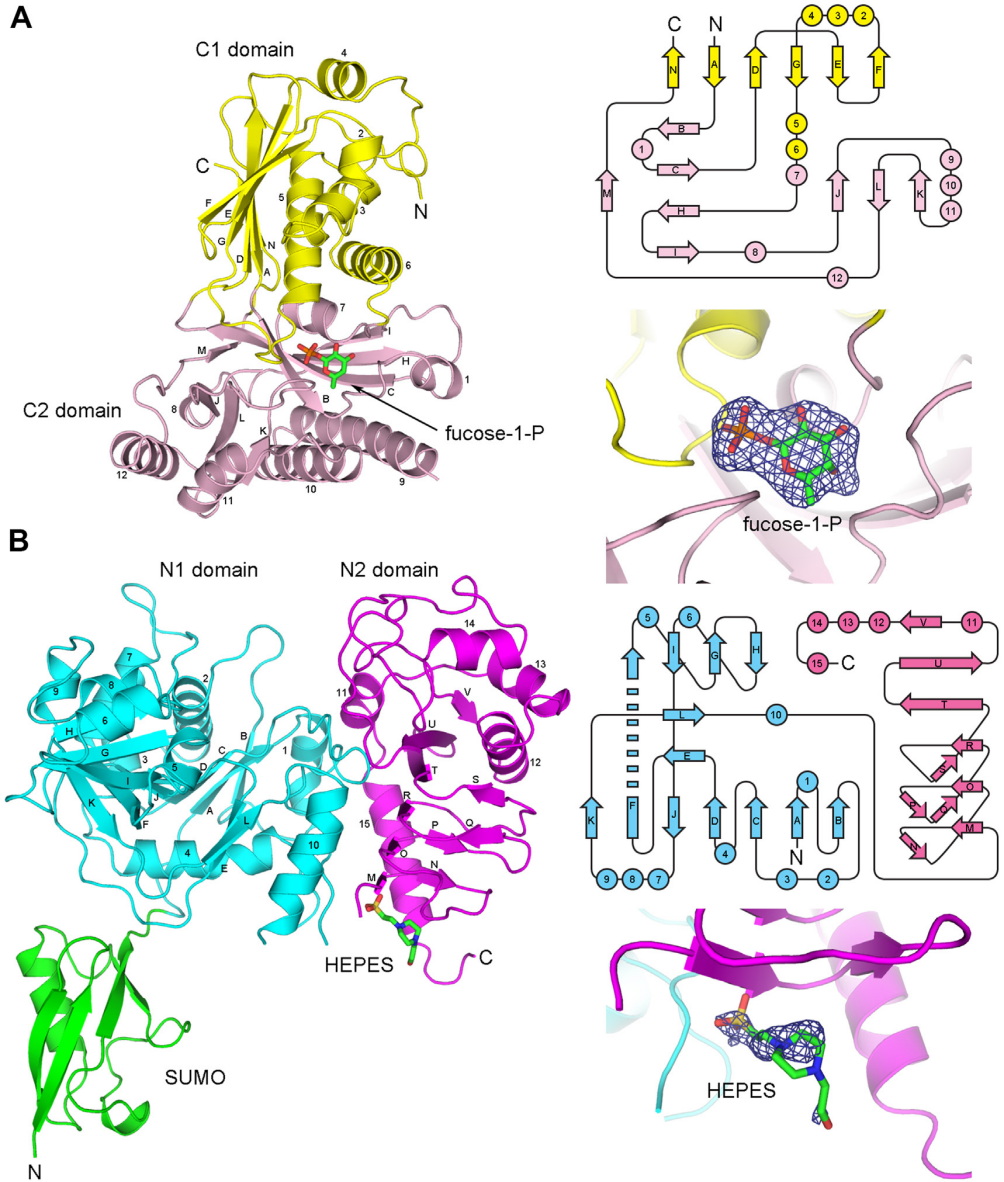
**Structures of FKP-CTD and SUMO-FKP-NTD.** The protein models are shown as *ribbon drawings* on the *left side*, which are accompanied by topology drawings on the *right*. *A*, the protein of FKP-CTD is folded into two domains C1 and C2, colored *yellow* and *pink*. The bound fucose-1-P is also shown as a *stick model*. Strand M associates with strand C at the N-terminal end of strand H. *B*, the structure of SUMO-FKP-NTD can be divided into three domains (namely SUMO, N1, and N2), and colored *green*, *cyan*, and *magenta*, respectively. The Hepes molecule attached to the N2 domain is shown as a *stick model*. Strand F spans two sheets, associating with strands *I*, *J*, and *K*. The corresponding Fo–Fc omit maps, contoured at four-σ level for each ligand, are shown as mesh on the *right*. CTD, C-terminal domain; NTD, N-terminal domain.

To provide insights into the catalytic mechanism, we further carried out structural determination by X-ray crystallography. Initial trials with both FKP-FL and its selenomethionine (Se–Met) derivative yielded an orthorhombic crystal form, which, however, did not contain the FL protein. Instead, it turned out to have a single FKP-CTD-like molecule as the asymmetric unit, probably a result of proteolysis. This crystal structure was solved by using multiple-wavelength anomalous diffraction method (Table S1). Subsequent soaking of the native crystals with fucose-1-P allowed direct observation of the binding mode of the sugar–phosphate product in the kinase active site. The refined FKP-CTD–fucose-1-P model to 2.46 Å resolution contains amino acid residues 571 to 812 and 831 to 949. The structure is similar to those of other sugar kinases (32, 33, 34, 35). FKP-CTD folds into two domains (Fig. 4*A*). The first domain (C1; 571–590, 623–756, and 943–949) consists of a six-strand, mostly an antiparallel β-sheet and five α-helices. The second domain (C2; 591–622, 757–942) comprises two (four- and three-strand) antiparallel β-sheets and seven α-helices. Fucose-1-P is bound between the two domains. Meanwhile, a new monoclinic crystal form that contained the intact FKP-FL was obtained under a different condition. It required the presence of GTP, which would bind to and stabilize the N-terminal pyrophosphorylase part, in the crystallization solution. By molecular replacement, two FKP-CTD monomers were successfully located. However, the disposition of FKP-NTD and the linker remained unknown.

To determine the FKP-NTD structure, we successfully crystallized the fusion protein of SUMO-FKP-NTD as well as its Se–Met derivative and used single-wavelength anomalous diffraction method for phasing (Table S2). The native structure was refined to a resolution of 2.35 Å. One SUMO-FKP-NTD monomer constitutes the asymmetric unit of this crystal. The long N-terminal arm of SUMO, the regions of 98 to 103 and 291 to 304 in FKP-NTD, and the four C-terminal residues were not included in the refined model. The part of FKP-NTD folds into two domains (Fig. 4*B*). The first domain (N1; 1–290) contains a seven-strand mixed-type β-sheet flanked by a four-strand antiparallel β-sheet on one side and a two-strand β-ribbon on the other side, plus 10 α-helices. The structure is similar to those of other nucleotidyl-sugar pyrophosphorylases (36, 37, 38, 39). The second domain (N2; 305–492) contains a three-turn left-handed β-helix, which is terminated by a two-strand β-ribbon and followed by a cap subdomain of a flanking β-strand and five α-helices. On the N-terminal side of the β-helix, some features of the electron density map suggested the presence of a bound ligand, which was modeled as a Hepes. The SUMO tag extends from the N terminus and has few interactions with FKP-NTD. However, in the crystal, it associates with the N1 domain of a dyad symmetry–related molecule, forming a dimer-like structure, which is different from that in the FKP-FL tetramer (see later and Fig. S3).

**Figure 5:**
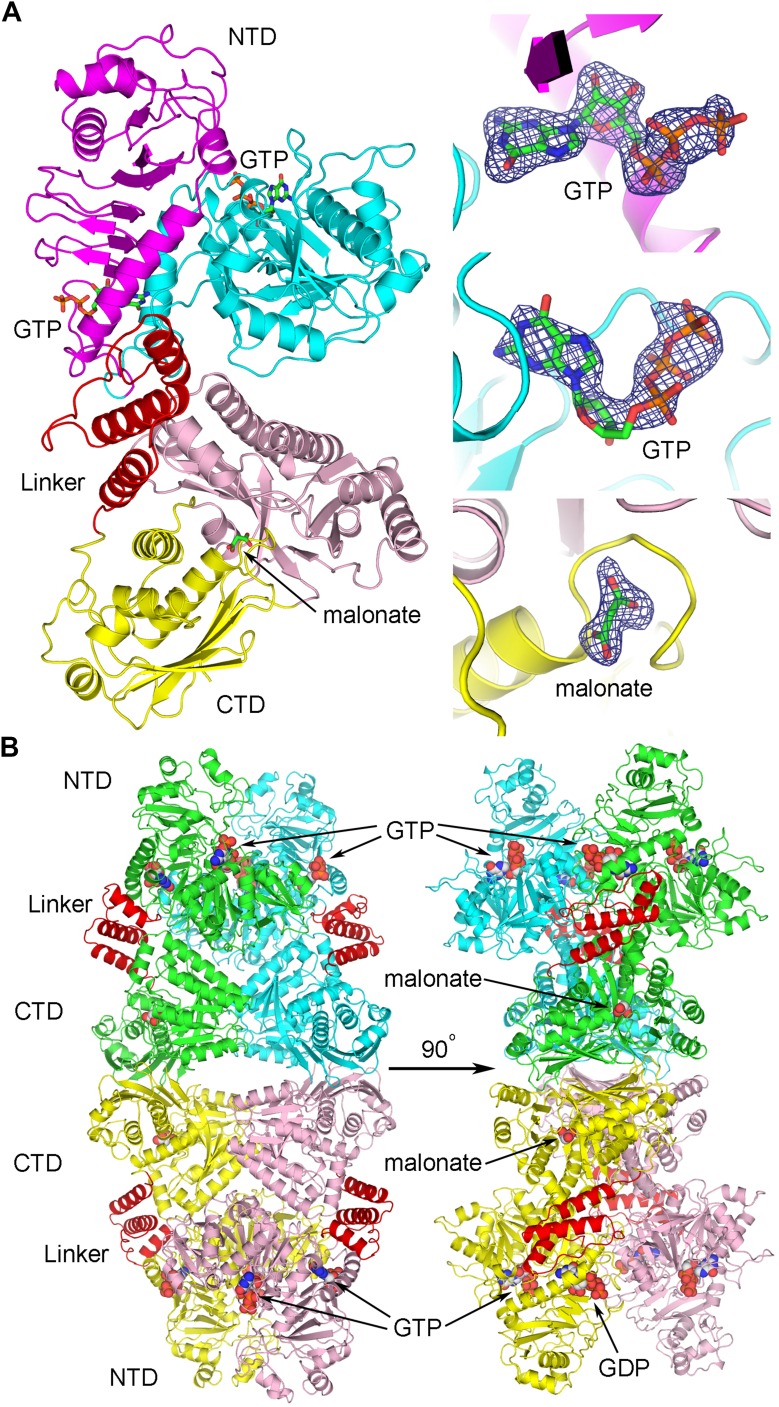
**Structure of FKP-FL.***A*, one subunit is shown as a *ribbon model* and colored in a similar way as in Figure 4, whereas the linker with three α-helices is in *red*. The bound ligands are shown as *sticks*. The corresponding Fo–Fc omit maps are contoured at four-σ level for each ligand and shown as mesh on the *right*. *B*, the tetramer is shown in two orthogonal views. The protein subunits are colored *green*, *cyan*, *yellow*, and *pink*, except for the linkers, which are *red*. The bound ligands are shown as *spheres*. FL, full length.

Using the FKP-NTD structure as the second search model, the complete crystal structure of FKP-FL was then determined also by molecular replacement, to a resolution of 2.3 Å. The monoclinic crystal contained two FKP-FL protomers in its asymmetric unit, and the twofold noncrystallographic symmetry relationship helped the model construction and structural refinement. All missing loops in the structures of FKP-CTD and FKP-NTD, as well as the linker, were seen in both protomers, including an additional α-helix (816–827) in FKP-CTD. The linker turned out to contain three α-helices (Fig. 5*A*). It constitutes a compact subdomain and appears to be more closely associated with the kinase domain than the pyrophosphorylase domain of the bifunctional protein. Two GTP molecules were found in the FKP-NTD region of one protomer. The other protomer had one GTP and one GDP. The first GTP corresponds to the nucleotide substrate of the pyrophosphorylase. The second GTP (or GDP) binds to the same site for Hepes in SUMO-FKP-NTD. Because of the use of Tacsimate in crystallization and glycerol in data collection, the electron density maps also suggested the presence of other bound ligands, such as malonate, acetate, and glycerol in various places. However, only a few could be modeled with certainty. The data collection and refinement statistics for all three crystals are summarized in Table S3.

### Ligand-binding modes of FKP

**Figure 6:**
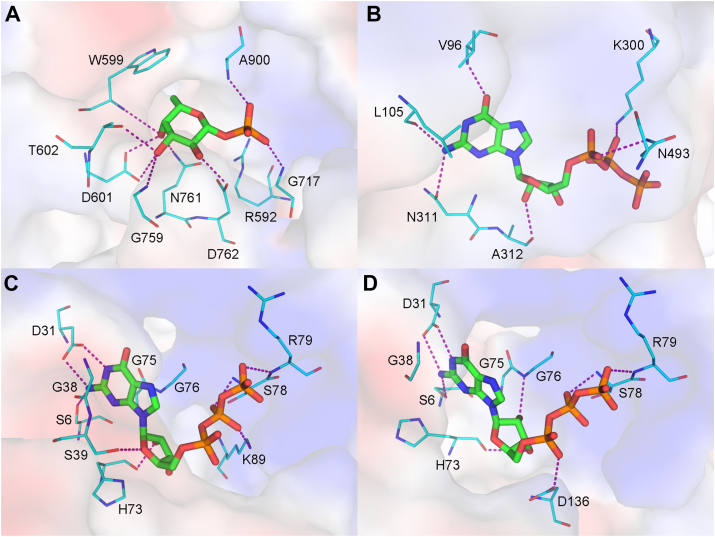
**Ligand-binding interactions of FKP.** The ligands are shown as *thick stick models* with carbon atoms colored in *green*. The protein residues are shown as *thin sticks* with *cyan carbons*. Corresponding electrostatic potential surfaces are shown in the background. Potential hydrogen bond formations are indicated by *dashed lines*. The ligands are (*A*) the fucose-1-P bound to the kinase active site of FKP-CTD; (*B*) the GTP bound to the noncatalytic site of FKP-FL; (*C*) the GTP bound to the pyrophosphorylase active site of one subunit in FKP-FL; and (*D*) the GTP bound to the active site of another subunit in FKP-FL. CTD, C-terminal domain; FL, full length.

The structures of the ligand–protein complexes are analyzed in detail to show specific interactions for the substrate binding and catalysis. Meanwhile, several mutants of FKP-FL were designed, expressed, and purified to determine their kinetic activities (Table 1). The reaction starts with the binding of l-fucose and ATP to the kinase active site. The bound product fucose-1-P in the complex crystal of FKP-CTD revealed the binding mode of l-fucose (substrate) because the structure of the sugar moiety, including β-configured C1-OH, is virtually identical. In the crystal, all free hydroxyl groups of fucose-1-P are involved in direct interactions with the protein (Fig. 6*A*). C2-OH is hydrogen bonded to the side chain of Asp762, C3-OH to those of Asp601 and Thr602, and C4-OH to Asp601 and Gln761. The side-chain indole group of Trp599 lies parallel to the plane of C4–C5–C6 at distances of 3.5 to 4.0 Å, apparently engaged in strong van der Waals or stacking interactions with the sugar moiety. The *k*_cat_/*K*_*m*_ values of W599A and D601A for fucose were reduced by more than 3000-fold, but their *K*_*m*_ for ATP remained similar. Other weaker interactions are also observed. For example, the anomeric O1 is 3.3 Å from both side chains of Arg592 and Asp762, and C3-OH and C4-OH are close to the backbone N of Gly759 and Trp599, respectively, at distances of 3.3 and 3.4 Å. In addition, two direct hydrogen bonds are formed between the phosphate group of fucose-1-P and the backbone N of Gly717 and Ala900. The inactive mutants E751A and D762A indicated the importance of these two carboxylate side chains in the catalysis, as both are conserved in the known homologous kinases (32, 33, 35).

The Hepes on the N-terminal side of the β-helix in the SUMO-FKP-NTD crystal was loosely bound. Except for the sulfonate part, it had few interactions with the protein, suggesting a possibility of nonspecific binding (Fig. S4*A*). This ligand-binding site is far from the expected active site of pyrophosphorylase, but later, the FKP-FL–GTP complex structure turned out to contain a GTP (or GDP) molecule in the same place (Figs. 6*B*; S4*B*). The guanine base is directly hydrogen bonded to the backbone N of Val96 *via* its O6 atom and to the backbone O of Leu105 and the side-chain OD1 of Asn311 *via* its N2 atom. The ribose O2′ is hydrogen bonded to the backbone O of Ala312. The α- and β-phosphate groups also form direct hydrogen bonds to the side chains of Asn493 and Lys300, respectively. GDP has a very similar binding mode, but Lys300 turns to bind to α-phosphate also, from which Asn493 moves a bit farther (Fig. S4*C*). In retrospect, the presence of GTP–GDP in the solution might have stabilized the protein structure, probably by binding to FKP-NTD, and thus allowed crystallization of FKP-FL. However, the function of this binding site remains a mystery. One possibility is to provide allosteric regulation, of which the mechanism needs further studies to be elucidated.

The bound GTP in the pyrophosphorylase active site is closely associated with the N1 domain. The guanine base is sandwiched between the planar peptide groups of Gly38 and Gly75 on one side and another (Fig. 6*C*). The side chain of Asp31 makes two hydrogen bonds to the N1 and N2 atoms of the base, whose N2 atom is also hydrogen bonded to the side chain of Ser6. Removal of the carboxylate group of residue 31 resulted in the almost inactive mutant D31A (Table 1). The ribose O3′ and O4′ make hydrogen bonds to the backbone O of His73 and the side chain of Ser39 in one monomer (Fig. 6*C*). Although the imidazole group of His73 is not involved in substrate binding, the *k*_cat_ values of mutant H73A for both substrates were reduced to less than 10%, suggesting some other roles of the side chain in catalysis. The β and γ-phosphate groups are hydrogen bonded to the backbone N of Ser78 and Arg79, respectively. Arg80, a highly conserved residue in FKPs from different species, was not observed to interact directly with the bound GTP in the FKP-FL crystal. However, the positively charged guanidinium group was close to the negatively charged pyrophosphate group at distances of 3.4 to 4.3 Å, likely to facilitate dissociation of the pyrophosphate from GTP, because the mutant R80A lost almost all its activity. Arg80 may also participate in fucose binding. The β-phosphate of GTP is also salt-bridged to the side chain of Lys89. In the other FKP-FL monomer, the GTP-binding mode differs slightly owing to fewer strong interactions (Figs. 6*D*; S4*D*). The ribose does not interact with Ser39 but with Gly76 instead, whose backbone N is now hydrogen bonded to the ribose O2′ atom. The α-phosphate also comes to interact with the side chain of Asp136. In addition, there are a number of other ligands in the FKP-FL crystal. Some of them are bound to the active sites and therefore are informative of substrate binding modes, as will be discussed in the next subsection.

### Structural comparison and computational modeling

The cryo-EM structure of FKP-FL has been reported (30). Although the tetrameric arrangement is identical to that observed in our crystal structure (Fig. 5*B*), the two structures show an overall RMSD of 4.1 Å for 2502 matched pairs of Cα atoms (Fig. S5*A*). The FKP-CTD part fits better with RMSD of 2.6 Å for 1392 Cα pairs, but the FKP-NTD part does not fit it at all (RMSD = 6.7 Å for 310 Cα in one subunit; Fig. S5*B*). Interestingly, an analysis of the packing in the F222 crystal of FKP-CTD also shows the same tetrameric organization despite its appearance as dimers in solution. This crystallographic tetramer deviates from that of FKP-FL by an RMSD of 1.2 Å for 1436 Cα pairs (Fig. S5*C*). The buried surface area in a separate dimer is 1230 Å^2^ on each FKP-CTD subunit, which is much larger than the 490 Å^2^ area buried further by tetramer formation. On the other hand, the NTD part contributes much larger interface areas to the formation of an FKP-FL tetramer, with 1110 Å^2^ surface buried on each subunit. The smaller interface area of 490 Å^2^ accounts for not only the observation of FKP-CTD dimers in solution but also the higher cooperativity of the fucokinase activity (Hill coefficient h = 2.0; Table 1), as there are fewer restraints for FKP-CTD to interact with one another, possibly *via* concerted conformational change upon substrate binding. In comparison, the additional restraints in FKP-FL imposed by the NTD part with the additional interface of 1110 Å^2^ may restrict the conformational changes in the CTD part of FKP-FL, resulting in the lower cooperativity (h = 1.4; Table 1).

**Figure 7:**
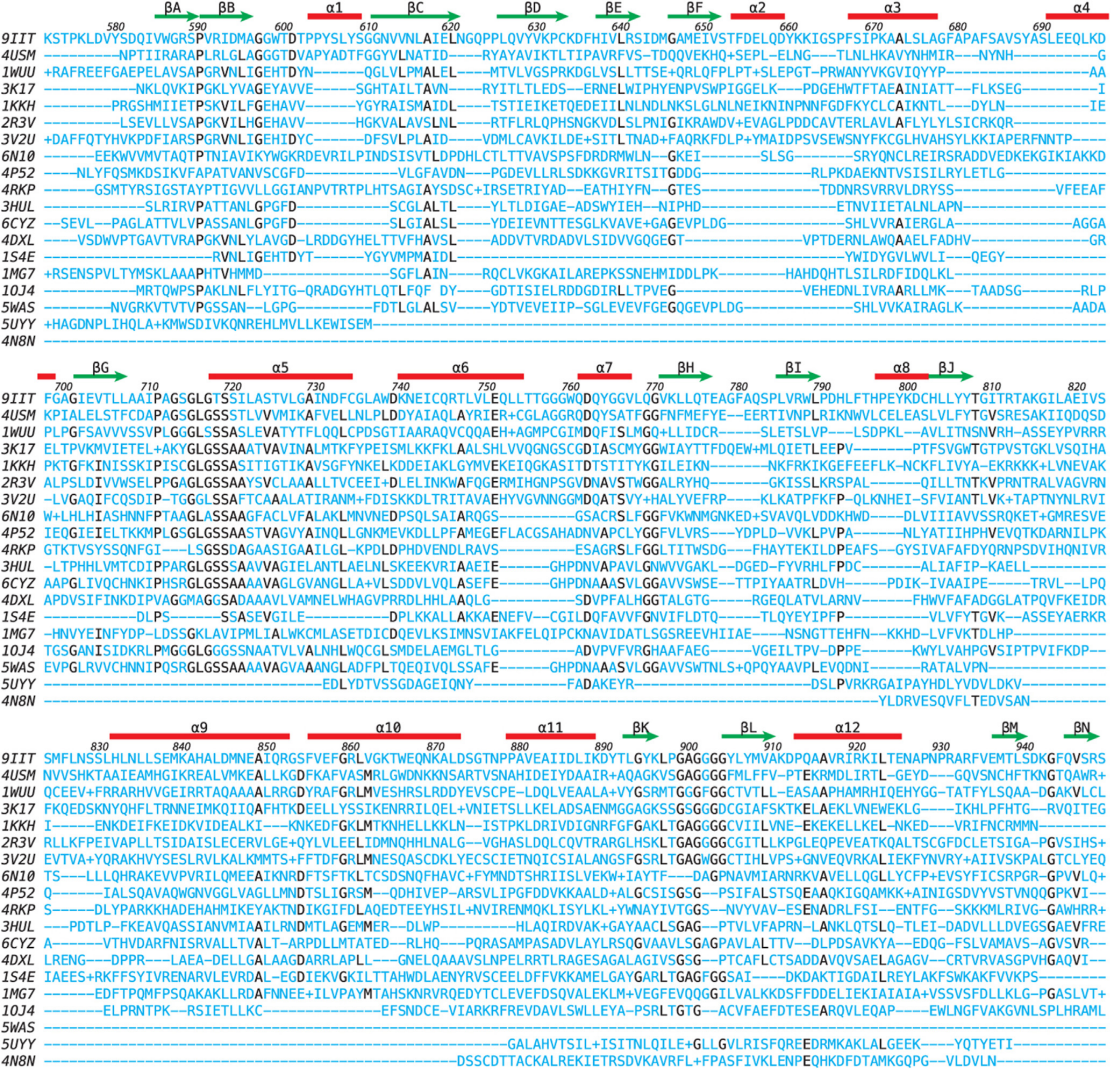
**Sequence comparison of FKP-CTD with similar proteins.** The amino acid sequence of FKP-CTD is aligned with those of the 18 structural homologs found by DALI. On the *left* are the PDB codes of the sequences. See Table S4 for additional information. On the *top* are the locations of the secondary structural elements in FKP-CTD, for which the amino acid residues are also numbered. Each *dash* (−) indicates one missing residue, whereas a plus sign (+) indicates the presence of more than one residue, that is, an insertion, at that location. Identical residues found in at least seven proteins are highlighted in *black*. CTD, C-terminal domain; PDB, Protein Data Bank.

A DALI search with FKP-CTD showed 17 other homologous structures than 5YYS in the Protein Data bank (PDB25) subset with Z scores above 10 (Table S4; Fig. 7). These include galactokinase, homoserine kinase, mevalonate kinase, and other sugar kinases, indicating that FKP-CTD belongs to the GHMP kinase family. The human galactokinase in complex with Mg-AMPPNP and galactose (1WUU) was selected for structural comparison and superimposed with FKP-CTD by an RMSD of 2.2 Å for 288 pairs of Cα atoms (34). The bound sugar in 1WUU matches well with the fucose-1-P in FKP-CTD and a glycerol in FKP-FL (Fig. S6*A*). Besides, the bound nucleotides and Mg^2+^ ions enable us to predict the binding modes of their equivalents in FKP, where a malonate and a glycerol were also observed in the FKP-FL crystal, in correspondence to the phosphates and the base, respectively. A model of FKP complex with fucose, ATP, and Mg^2+^ was constructed. As shown in Fig. S6*B*, the phosphate groups along with Mg^2+^ (or other metal ions) bind to the region 712 to 720, which comprises the N terminus of an α-helix and its preceding loop. The base is backed by the side chains of Ile669 and Leu722 and covered by Met645. It probably makes hydrogen bonds with the side chain of Asp644 and the backbone of Pro666. The ribose, however, shows few specific interactions.

**Figure 8:**
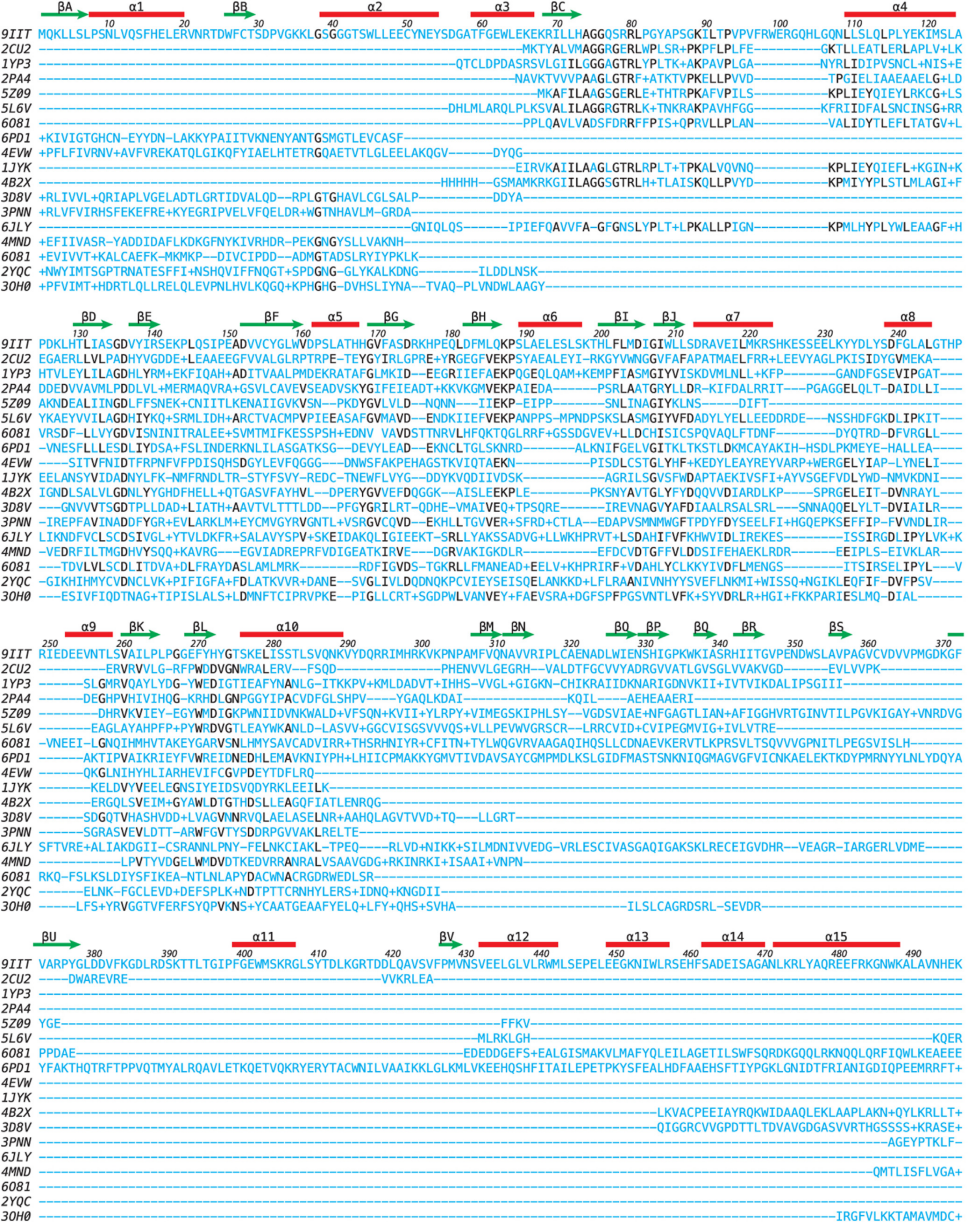
**Sequence comparison of FKP-NTD with similar proteins.** Here the sequences of 17 structural homologs are aligned. See Table S5 for additional information. Identical residues found in at least five proteins are highlighted in *black*. NTD, N-terminal domain.

Another DALI search with FKP-NTD showed 13 homologous structures in the PDB25 subset with Z scores above 10 (Table S5; Fig. 8), including 5YYS, mannose-1-phosphate guanylyltransferase, glucose-1-phosphate adenylyltransferase, and other sugar–nucleotide pyrophosphorylases. Interestingly, two translation initiation factor subunits suggested that FKP-NTD is related to the other sugar-1-phosphate nucleotidylyltransferases, although with lower Z scores and sequence identity. Here, a bacterial UDP-glucose pyrophosphorylase in complex with Mg^2+^ and UDP-glucose (2PA4) was chosen for comparison and superimposed with FKP-NTD by an RMSD of 3.2 Å for 168 Cα pairs. The nucleotide part of UDP-glucose in 2PA4 matches well with the bound GTP in FKP-FL (Fig. S7*A*). The C2–C3–C4 moiety of glucose in 2PA4 also has an equivalent glycerol in FKP-FL. The bound Mg^2+^ from 2PA4 allowed us to model the metal ions into their binding sites in FKP, which may involve the side chains of Asp136 and His272 (Fig. S7*B*). In a fucose-1-P model constructed based on the glucose and glycerol positions, the methyl group is facing the nonpolar side chains of Val137 and Phe270. The free hydroxyl groups may interact with the side chains of Gln186 and His168 as well as the backbone of Gly169. The phosphate may interact with the side chains of Lys187 and His272 (Fig. S7*B*). Removal of the side chains of His168, Lys187, and His272 should disturb the interactions with the fucose and phosphate parts of fucose-1-P as indicated by the 13- to 30-fold reduction of binding affinity for fucose in the corresponding mutants (Table 1).

On the other hand, a left-handed β-helix is found as a C-terminal component in FKP-NTD and several other sugar–nucleotide pyrophosphorylase homologs (Table S5). β-Helical fold proteins were found in many microbial pathogens (40) and in all sorts of nonpathogens in straightforward metabolic enzymes. Among the aforementioned homologs, the bacterial enzymes *Thermus thermophilus* mannose-1-phosphate guanylyltransferase (2CU2) and *Sulfolobus tokodaii* sugar-1-phosphate nucleotidylyltransferase (5Z09), and the plant enzyme *Solanum tuberosum* (potato) ADP-glucose pyrophosphorylase (1YP3) are each associated into dimer, trimer, and tetramer, respectively (Fig. S8). However, the β-helix in FKP (which is also a bacterial enzyme) does not participate directly in oligomer formation. Instead, it appears to serve as a stable folding unit that sustains the helices and loops in the N2 domain, which constitutes parts of the substrate-binding site for fucose-1-P and the nonsubstrate-binding site for GTP–GDP.

### Mechanism of FKP catalysis

**Figure 9:**
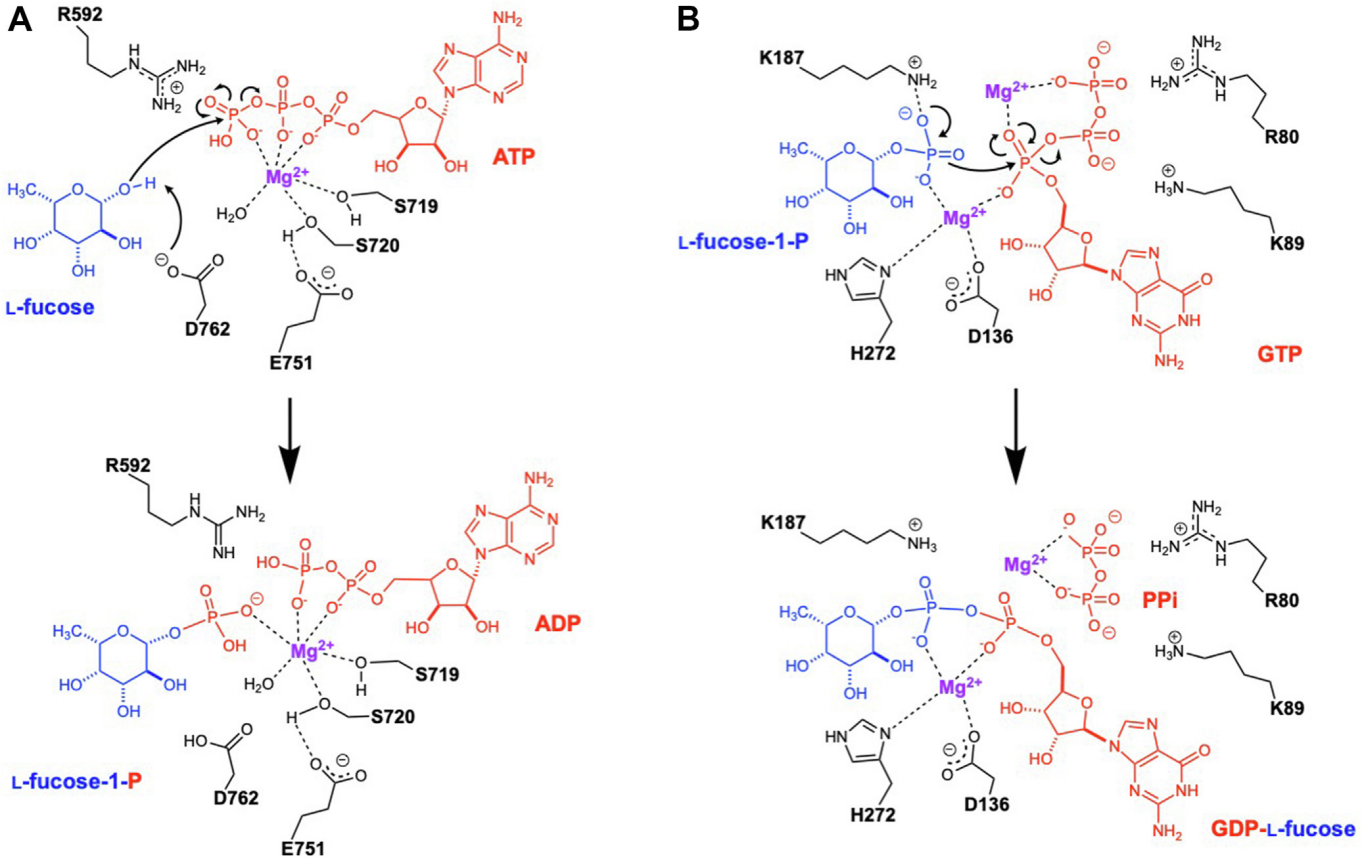
**Proposed catalytic mechanism for FKP.** Schematic drawings show the chemical reactions, substrates, products, and major participating amino acid residues in (*A*) for fucokinase and (*B*) for GDP-fucose pyrophosphorylase.

The catalytic mechanism of galactokinase and other GHMP kinase family enzymes has been well studied (41). In general, the sugar substrate binds to the enzyme first, and it does not require the prior presence of ATP in the active site. All interactions between l-fucose (or other similar substrate) and FKP may form before the ATP binding (Fig. 9*A*). When ATP enters the active site, probably as a complex with Mg^2+^ or another divalent metal ion, the triphosphate moiety is bound by the glycine-rich loop 712 to 720. The metal ion is coordinated not only to the phosphates but also to the side chain of Ser719 and two waters fixed by Ser720 and Asp762 and maybe an additional water molecule in case of Ca^2+^ or Mn^2+^. The reaction starts with the side-chain carboxylate of Asp762 that serves as a general base to subtract the proton from C1-OH of the bound fucose (Fig. 9*A*), enhancing the nucleophilicity to attack the phosphorous atom of the γ-phosphate of the bound ATP. The pentavalent intermediate is stabilized by the backbone NH groups of the glycine-rich loop, the side chain of Arg592, and the metal ion. The γ-phosphate is then hydrolyzed from ATP, resulting in the formation of ADP, and the reaction is completed by protonation of the β-phosphate and deprotonation of Asp762, probably mediated by solvent. Glu751 is too far to reach the metal ion in FKP without significant conformational changes, but the equivalent in PDB 1WUU binds directly to it (34). We also noted a 6°–9° rotation of the C2 domain with respect to the C1 domain of FKP when compared with 1WUU. The role of this conformational change in catalysis and cooperativity remains to be further investigated.

Regarding the subsequent reaction catalyzed by pyrophosphorylase, whether fucose-1-P or GTP binds first is an open question. GTP should come in along with 2 Mg^2+^ ions, as this class of sugar nucleotidyltransferases shares a similar two-metal-mediated mechanism B for the pyrophosphate elimination, which is distinct from that of DNA–RNA polymerases (42). The first Mg^2+^ is bound to the side chains of Asp136 and probably His272, the phosphate of fucose-1-P, the α-phosphate of GTP, and two or three coordinating water molecules. The second Mg^2+^ is bound to the other side of the α-phosphate of GTP, four or five water molecules, and probably the γ-phosphate (Fig. 9*B*). The reaction starts with the nucleophilic attack by the phosphate of fucose-1-P toward the α-phosphate of GTP, which is facilitated by the Lys187 side chain (Fig. 9*B*). The negative charge is thus developed on the α-phosphate of GTP after the attack is stabilized mainly by the two Mg^2+^ ions, but eventually relocated to the β-phosphate, which is facilitated by the interactions with the side chains of Arg80 and Lys89 and the backbone NH group of Ser78. It is proposed that the first Mg^2+^ ion likely remains bound to the phosphate groups of GDP-fucose, whereas the second Mg^2+^ may migrate to coordinate with the original β- and γ-phosphate groups, leading to the formation of pyrophosphate. The resulting pyrophosphate complex leaves the active site as a complex with Mg^2+^, probably assisted by the adjacent side chains of Arg80 and possibly Arg79. There are also significant conformational differences between the N-terminal part of FKP-FL and SUMO-FKP-N. Presumably, the domain flexibility facilitates the substrate binding and product release.

Finally, no potential tunnel can be identified for trafficking the intermediate product, fucose-1-P, between the active sites of kinase and pyrophosphorylase. The 70-residue linker seems only to serve as a connection between the two enzyme domains. To produce GDP-fucose from fucose, ATP, and GTP, perhaps the two-step reaction is made more efficient just by tying the two enzyme domains together.

In summary, we not only determined the crystal structures of the bifunctional enzyme FKP as separate N- and C-terminal parts and as a FL protein but also characterized a number of their biochemical properties, such as pH dependence, metal ion requirement, substrate specificity, and kinetic parameters. By analyzing the ligand-bound crystal structures and comparison with other related enzyme structures, reasonable substrate-binding modes and catalytic pathways are proposed for both the fucokinase and pyrophosphorylase domains of FKP. These findings will pave the way for complete elucidation of FKP catalytic mechanism, a unique enzyme that directly converts l-fucose into GDP-β-l-fucose. Notably, both enzyme domains show a relaxed specificity regarding the C5-substituents, as the methyl group of fucose or fucose-1-P is accommodated by a spacious pocket in each active site but not engaged in specific interaction with the protein atoms. The crystal structures will thus serve as a firm basis for protein engineering to efficiently generate desired sugar–nucleotide products, which can find wide applications in the field of carbohydrate research.

## Experimental procedures

### Gene cloning and protein expression and purification

The *fkp* gene was amplified from the genomic DNA of *B. fragilis* 9343 (GenBank accession number: AY849806). The wildtype *fkp* and chimeric genes were then cloned into a pET-16b vector or a pET SUMO vector. The desired constructs were validated by DNA sequencing and transformed into *Escherichia coli* BL21 (DE3) strain for protein expression. The transformed bacteria were cultured at 37 °C until the absorbance reached 0.6 at 600 nm and then induced by addition of isopropyl-1-thio-*β*-D-galacto-pyranoside to a final concentration of 0.2 mM. Bacteria were harvested by centrifugation after an incubation of 20 h at 16 °C. The pellets were lysed by cell disruption system (Constant TS 0.75 kW) at 20,000 psi and ultracentrifuged at 125,000*g* for 1 h at 4 °C to separate the soluble proteins from the crudes. The clarified supernatant was applied to a HiTrap chelating HP column (GE Healthcare) according to the instruction manual. The column was washed with the solution containing 50 mM Na_2_HPO_4_, 10 mM KH_2_PO_4_, 500 mM NaCl, 10% glycerol, and 20 mM imidazole (pH 7.4). The bound protein was then eluted by linear gradient of the same buffer containing 400 mM imidazole. The peak fractions were collected and further purified by gel filtration in a HiLoad 16/600 Superdex 200 pg column (GE Healthcare) in the same buffer without imidazole. The purified FKP was concentrated to 20 to 30 mg/ml in an Amicon Ultra-30 concentrator (Millipore) and stored at −80 °C. All the FKP mutant proteins were expressed and purified as described for the wildtype protein. The Se–Met-labeled FKP was produced by using an Overnight Express Autoinduction System 2 (Novagen) and purified in a similar manner. The protein concentration was determined using the Bradford method using bovine serum albumin as a standard.

### AUC analysis

The SV of FKPs was measured by AUC in a Beckman-Coulter XL-A ultracentrifuge at 20 °C. The proteins were diluted with 50 mM Tris (pH 8.0), 150 mM NaCl to a concentration of about 0.5 mg ml^−1^. Samples were loaded into standard double sector cells with an Epon charcoal-filled centerpiece, mounted in a four-hole An-60 Ti rotor, and centrifuged at 40,000 rpm. The buffer density, viscosity, and partial specific volume of proteins were predicted by using the SEDNTERP program. The cells were scanned at 280 nm in a continuous mode, and the experimental data were analyzed by the Sedfit program (version 14.1; http://sedfitsedphat.nibib.nih.gov/software). After the ultracentrifugation, the protein sample was visually checked for clarity, and no indication of precipitation was found.

### SEC–MALS analysis

The absolute MW of FKPs was determined by static light scattering using a miniDAWN TREOS and an Optilab T-rEX differential refractive index detector (Wyatt Technology) coupled to an Agilent 1260 Infinity HPLC with an ENrich SEC 650 column (Bio-Rad). Bovine serum albumin was used for system calibration before applying the target protein into SEC–MALS. FKPs (1 mg ml^−1^) were used for the SEC–MALS analysis with a running buffer containing 50 mM Tris (pH 8.0) and 150 mM NaCl under a flow rate of 0.5 ml min^−1^. The data were analyzed using ASTRA 6 software (Wyatt Technology) with the dn/dc value set to 0.185 ml g^−1^.

### Substrate-specificity study

To study the substrate specificity of FKP, a number of monosaccharides were examined, including l-fucose, d-arabinose, l-arabinose, d-allose, l-galactose, d-glucose, l-glucose, d-mannose, d-lyxose, d-ribose, d-xylose, l-xylose, 5-azido-l-fucose, and 5-alkynyl-l-fucose. An enzyme reaction mixture was incubated at 37 °C for 3 h, contained 5 mM monosaccharide, 5 mM ATP, 5 mM GTP, 5 mM MgCl_2_, FKP, and inorganic pyrophosphatase (to hydrolyze the side product, pyrophosphate). The reactions were terminated by heating in a boiling water bath for 1 min. Product conversions were analyzed by thin layer chromatography with *p*-anisaldehyde staining.

### Enzyme activity assay

The GDP-fucose pyrophosphorylase activity was measured using the EnzCheck Pyrophosphate Assay Kit (Invitrogen). The reaction mixture contained 50 mM Hepes (pH 7.5), 2 mM MgCl_2_, 200 μM 2-amino-6-mercapto-7-methylpurine ribonucleoside, 0.05 U nucleoside phosphorylase, 0.012 U inorganic pyrophosphatase, GTP, fucose-1-P, and FKP (or chimeric FKP). The reaction progress was detected by an increase in an absorbance at 360 nm. The l-fucokinase activity was determined by coupling the enzyme reaction with pyruvate kinase–lactate dehydrogenase to monitor the consumption of NADH with the decrease in absorbance of 340 nm. The reactions contained 50 mM Hepes (pH 7.5), 2 mM MnCl_2_, 1.5 mM PEP, 500 μM NADH, 5 U pyruvate kinase, 5 U lactate dehydrogenase ATP, l-fucose, and FKP (or chimeric FKP). The resulting data were transferred to GraphPad Prism software and fitted either by Michaelis–Menten equation to obtain kinetic parameters or Hill equation to determine the cooperative coefficient. The units of *K*_*m*_ and *k*_cat_ are shown as mM (*mini*molar) and sec^−1^ (per second), respectively.

### Crystallization, X-ray data collection, and structural determination

The purified proteins were concentrated to ∼15 mg ml^−1^ for crystallization screen of over 960 different conditions at the sitting-drop vapor-diffusion plates. The initial crystallization condition was further refined manually. The protein solution was mixed with equal volumes of crystallization buffer at 22 °C *via* the hanging-drop vapor-diffusion method. Finally, FKP-NTD (amino acids 1–496) fused with SUMO tag protein, as well as its SeMet derivative, was crystallized in 0.1 M Hepes (pH 7.5), 0.2 M ammonium acetate, and 18% PEG3350. FKP-CTD was formed and further crystallized through FKP autodegradation and further crystallized within few days in 50 mM Hepes (pH 7.0), 0.8% trypton, and 10% PEG3350. The complex crystals of FKP-CTD were obtained by soaking the apo-form crystals in a reservoir solution containing either 10 mM fucose-1-P and 3 mM MnCl_2_ or 10 mM l-fucose. Finally, FKP-FL was successfully crystallized by using 8% Tacsimate (pH 7.0) and 20% PEG3350, with the required presence of 15 mM GTP and 15 mM MgCl_2_ in the mother liquor.

Before being mounted on the goniometer prechilled in a liquid nitrogen gas system, the crystals were immersed in reservoir solution supplemented with 30% (v/v) glycerol as a cryoprotectant. Initial crystal diffraction-quality screening was performed at the home laboratory FR-E + SuperBright X-ray diffraction system equipped with an R-AXIS HTC detector (Rigaku). High-resolution diffraction data were collected at BL13B and BL15A1 of the National Synchrotron Radiation Research Center and at 44XU of SPring-8. The data were processed using HKL2000 program package (43). The PHENIX (44), REFMAC5 (45), and COOT (46) programs were used for solving the phase of the structure, molecular replacement, structural refinement, and manual adjustment of the models. All structural figures were prepared in PyMOL (Schrӧdinger, LLC) and Discovery Studio (Dassault Systèmes) software.

## Data availability

All the data are contained within the article, except for the three X-ray structures, the details of which are available in the PDB database. Please refer the PDB ID numbers of 9IIP (for FKP-CTD/fucose-1-P), 9IIS (for SUMO-FKP-NTD), and 9IIT (for FKP-FL/GTP).

## Supporting information

This article contains supporting information.

## Conflict of interest

The authors declare that they have no conflicts of interest with the contents of this article.

## Supporting information

Supporting information

**Abbreviations**
AUC, analytical ultracentrifugation
CTD, C-terminal domain
FKP, L-fucokinase/GDP-β-l-fucose pyrophosphorylase
FL, full length
fucose-1-P, l-fucose-1-phosphate
MW, molecular weight
NTD, N-terminal domain
PDB, Protein Data Bank
Se–Met, selenomethionine
SEC–MALS, size-exclusion chromatography–multiangle light scattering
SV, sedimentation velocity
