# Supplementary material for: Structural insight into the catalytic mechanism of the bifunctional enzyme l-fucokinase/GDP-fucose pyrophosphorylase
Source: J Biol Chem. 2025 Feb 22;301(4):108344. doi: 10.1016/j.jbc.2025.108344 (PMC11982464; doi:10.1016/j.jbc.2025.108344)
Supplement: Supporting information [file mmc1.docx]

**Table S1**. Data collection and MAD phasing statistics for the Se-FKP-CTD crystal.

|  | Peak | Inflection | High remote |
| --- | --- | --- | --- |
| Data collection |  |  |  |
| Wavelength (Å) | 0.97903 | 0.97922 | 0.96400 |
| Space goup | F222 | F222 | F222 |
| Unit-cell *a*, *b*  *c* (Å) | 95.05, 119.43  162.61 | 94.96, 119.30  162.45 | 94.79, 119.10  162.17 |
| Resolution (Å) ^a^ | 30-2.73 (2.83-2.73) | 30-2.69 (2.79-2.69) | 30-2.65 (2.74-2.65) |
| Unique reflections | 12460 (1238) | 13072 (1301) | 13570 (1349) |
| Completeness (%) | 99.8 (100) | 99.8 (100) | 99.8 (100) |
| Redundancy | 8.1 (8.2) | 8.1 (8.2) | 8.1 (8.0) |
| I/σ(I) | 22.4 (5.5) | 22.0 (5.3) | 47.1 (5.5) |
| R_merge_ | 0.067 (0.478) | 0.070 (0.494) | 0.068 (0.493) |
| MAD-Phasing |  |  |  |
| Number of Se sites |  | 9 |  |
| Figure of merit |  | 0.56 |  |

^a^ The numbers in parentheses are for the highest resolution shells.

**Table S2**. Data collection and SAD phasing statistics for the Se-SUMO-FKP-NTD crystal.

|  | Peak |
| --- | --- |
| Data collection |  |
| Wavelength (Å) | 0.97910 |
| Space goup | I222 |
| Unit-cell a, b, c (Å) | 86.29, 105.92, 141.31 |
| Resolution (Å) ^a^ | 30-2.49 (2.58-2.49) |
| Unique reflections | 21823 (2171) |
| Completeness (%) | 100 (100) |
| Redundancy | 14.7 (14.4) |
| I/σ(I) | 21.3 (2.8) |
| R_merge_ | 0.157 (1.261) |
| SAD-Phasing |  |
| Number of Se sites | 18 |
| Figure of merit | 0.41 |

^a^ The numbers in parentheses are for the highest resolution shells.

**Table S3.** Data collection and refinement statistics of the FKP crystals

|  | FKP-CTD/fucose-1-P | SUMO-FKP-NTD | FKP-FL/GTP |
| --- | --- | --- | --- |
| Data collection |  |  |  |
| Space group | F222 | I222 | C2 |
| Unit-cell *a*, *b*, *c* (Å) | 95.0, 120.8, 165.0 | 86.6, 109.8, 144.1 | 211.8, 96.1, 127.4 |
| β (°) |  |  | 97.1 |
| Resolution (Å) ^a^ | 30-2.46 (2.55-2.46) | 30-2.35 (2.43-2.35) | 30-2.3 (2.38-2.30) |
| Unique reflections | 17444 (1726) | 28528 (2831) | 111983 (11074) |
| Redundancy | 6.2 (6.2) | 4.9 (4.8) | 3.6 (3.4) |
| Completeness (%) | 99.6 (99.9) | 99.4 (99.3) | 98.8 (98.2) |
| Overall I/σ(I) | 29.9 (2.2) | 23.4 (2.1) | 22.8 (1.6) |
| Average CC_1/2_ | 0.942 (0.737) | 0.970 (0.893) | 0.918 (0.696) |
| R_merge_ (%) | 5.0 (96.6) | 6.5 (77.1) | 7.7 (94.0) |
| Refinement |  |  |  |
| No. of reflections | 17428 (1719) | 28436 (2721) | 110215 (10713) |
| Completeness (%) | 99.5 (99.0) | 98.8 (95.5) | 97.9 (95.4) |
| R-work | 0.186 (0.312) | 0.203 (0.295) | 0.200 (0.300) |
| R-free^b^ | 0.218 (0.358) | 0.254 (0.334) | 0.232 (0.348) |
| RMSD from ideal values |  |  |  |
| Bond lengths (Å) | 0.0021 | 0.0026 | 0.0021 |
| Bond angles (°) | 0.53 | 0.65 | 0.56 |
| Average B (Å^2^) / No. of atoms |  |  |  |
| Protein | 95.9 / 2775 | 93.5 / 4371 | 67.4 / 14914 |
| Ligands | 64.4 / 15 | 123.1 / 19 | 77.7 / 169 |
| Water | 88.0 / 187 | 82.8 /227 | 56.6 / 843 |
| Ramachandran statistics (%)^c^ |  |  |  |
| Favored | 95.8 | 94.3 | 97.1 |
| Allowed | 3.8 | 5.7 | 2.9 |
| Outliers | 0.6 | 0.0 | 0.0 |
| Clash score | 7.5 | 6.4 | 5.3 |
| MolProbity score | 2.17 | 2.16 | 1.56 |
| PDB code | 9IIS | 9IIP | 9IIT |

^a^ Numbers in parentheses are for the highest resolution shells.

^b^ Five percent of randomly selected data were set aside for calculating R-free.

^c^ The stereochemistry of each model was validated by using MolProbity (ref)

ref: Chen, V. B., Arendall, W. B., 3rd, Headd, J. J., Keedy, D. A., Immormino, R. M., Kapral, G. J. *et al.* (2010) MolProbity: all-atom structure validation for macromolecular crystallography. *Acta Crystal Sec D, Biol Crystal* **66**, 12-21; 10.1107/S0907444909042073

**Table S4**. DALI search results with the PDB25 subset and FKP-CTD (top 20).

| Chain | Z | rmsd | lali | nres | %id | Description |
| --- | --- | --- | --- | --- | --- | --- |
| 5yys-A | 42.5 | 1.8 Å | 368 | 811 | 84 | L-fucokinase |
| 4usm-A | 39.1 | 2.0 Å | 331 | 345 | 24 | Putative sugar kinase |
| 6q8z-A | 32.4 | 2.7 Å | 321 | 392 | 17 | Galactokinase |
| 3k17-A | 31.2 | 2.8 Å | 321 | 355 | 12 | LIN0012 protein |
| 1kkh-A | 29.3 | 3.2 Å | 306 | 317 | 18 | Mevalonate kinase |
| 6qje-D | 27.8 | 2.8 Å | 298 | 352 | 17 | Galactokinase |
| 2r3v-A | 26.4 | 4.5 Å | 313 | 390 | 15 | Mevalonate kinase |
| 3v2u-C | 25.5 | 2.8 Å | 325 | 516 | 18 | Galactose/lactose metabolism regulator |
| 6n10-A | 24.1 | 3.4 Å | 308 | 403 | 14 | Diphosphomevalonate decarboxylase |
| 4p52-A | 23.7 | 3.3 Å | 294 | 315 | 15 | Homoserine kinase |
| 4rkp-B | 23.6 | 3.1 Å | 291 | 320 | 13 | Putative uncharacterized protein TA1305 |
| 3hul-B | 21.7 | 3.0 Å | 264 | 269 | 16 | Homoserine kinase |
| 6cyz-A | 20.9 | 3.1 Å | 277 | 295 | 16 | Homoserine kinase |
| 4dxl-A | 20.8 | 3.8 Å | 275 | 304 | 14 | Diphosphocytidyl- metylerythritol kinase |
| 1s4e-G | 18.8 | 2.7 Å | 228 | 255 | 21 | Galactokinase |
| 1mg7-B | 17.9 | 3.2 Å | 258 | 352 | 10 | Early switch protein XOL-1 |
| 1oj4-A | 16.2 | 3.7 Å | 255 | 283 | 16 | Diphosphocytidyl- metylerythritol kinase |
| 5was-A | 13.0 | 2.8 Å | 168 | 180 | 17 | Homoserine kinase |
| 5uyy-A | 7.3 | 12.6 Å | 94 | 374 | 7 | Prephenate dehydrogenase |
| 4n8n-B | 6.9 | 2.5 Å | 70 | 103 | 6 | Cell division protein FtsX |
| (full PDB) |  |  |  |  |  |  |
| 1wuu-A | 30.2 | 2.5 Å | 304 | 391 | 17 | Galactokinase |

Note: PDB 6q8z, 6qje and 1wuu come from the same protein, human galactokinase.

**Table S5**. DALI search results with the PDB25 subset and FKP-NTD (top 20).

| Chain | Z | rmsd | lali | nres | %id | Description |
| --- | --- | --- | --- | --- | --- | --- |
| 5yys-A | 14.4 | 3.6 Å | 307 | 811 | 48 | L-fucose-1-P guanylyltransferase |
| 2cu2-A | 12.8 | 3.9 Å | 245 | 335 | 15 | Mannose-1-P guanylyltransferase |
| 5z09-E | 11.9 | 3.3 Å | 229 | 369 | 18 | Dual sugar-1-P nucleotidylyltransferase |
| 6o81-A | 11.7 | 3.4 Å | 263 | 590 | 13 | Translation initiation factor EIF-2B subunit |
| 1yp3-C | 11.3 | 3.0 Å | 239 | 435 | 18 | Glucose-1-P adenylyltransferase |
| 5l6v-P | 10.9 | 3.2 Å | 234 | 423 | 16 | Glucose-1-P adenylyltransferase |
| 5l6v-I | 10.9 | 3.2 Å | 227 | 400 | 16 | Glucose-1-P adenylyltransferase |
| 4evw-A | 10.9 | 3.4 Å | 181 | 244 | 9 | NDP-sugar pyrophosphorylase |
| 2pa4-B | 10.8 | 5.0 Å | 191 | 299 | 16 | UDP-glucose-1-P uridylyltransferase |
| 5l6s-N | 10.5 | 3.3 Å | 223 | 369 | 16 | Glucose-1-P adenylyltransferase |
| 6pd1-C | 10.4 | 4.3 Å | 183 | 617 | 10 | Nucleotidyltransferase/aminotransferase |
| 4b2x-B | 10.3 | 4.4 Å | 194 | 302 | 13 | Glucose-1-P thymidylyltransferase |
| 3d8v-A | 10.1 | 3.8 Å | 202 | 477 | 13 | Bifunctional protein GLMU |
| 1jyk-A | 9.9 | 3.1 Å | 172 | 229 | 13 | CTP:phosphocholine cytidylyltransferase |
| 6jly-E | 9.8 | 4.6 Å | 242 | 414 | 14 | Translation initiation factor EIF-2B subunit |
| 3pnn-A | 9.5 | 3.8 Å | 180 | 301 | 10 | Conserved domain protein |
| 4mnd-A | 8.7 | 3.8 Å | 191 | 408 | 8 | CTP:L-myoinositol-1-P cytidylyltransferase |
| 6o81-I | 8.5 | 3.7 Å | 168 | 240 | 15 | Translation initiation factor EIF-2B subunit |
| 3oh0-A | 7.9 | 4.1 Å | 205 | 585 | 8 | UDP-sugar pyrophosphorylase |
| 2yqc-A | 7.8 | 4.0 Å | 196 | 480 | 10 | UDP-GlcNAc pyrophosphorylase |

Note: PDB 5l6s and 5l6v come from the same protein, *E. coli* Glucose-1-P adenylyltransferase.
